# Supplementary figures and images for: The Aspergillus fumigatus transcription factor RglT is important for gliotoxin biosynthesis and self-protection, and virulence
Source: PLoS Pathog. 2020 Jul 15;16(7):e1008645. doi: 10.1371/journal.ppat.1008645 (PMC7384679; doi:10.1371/journal.ppat.1008645)

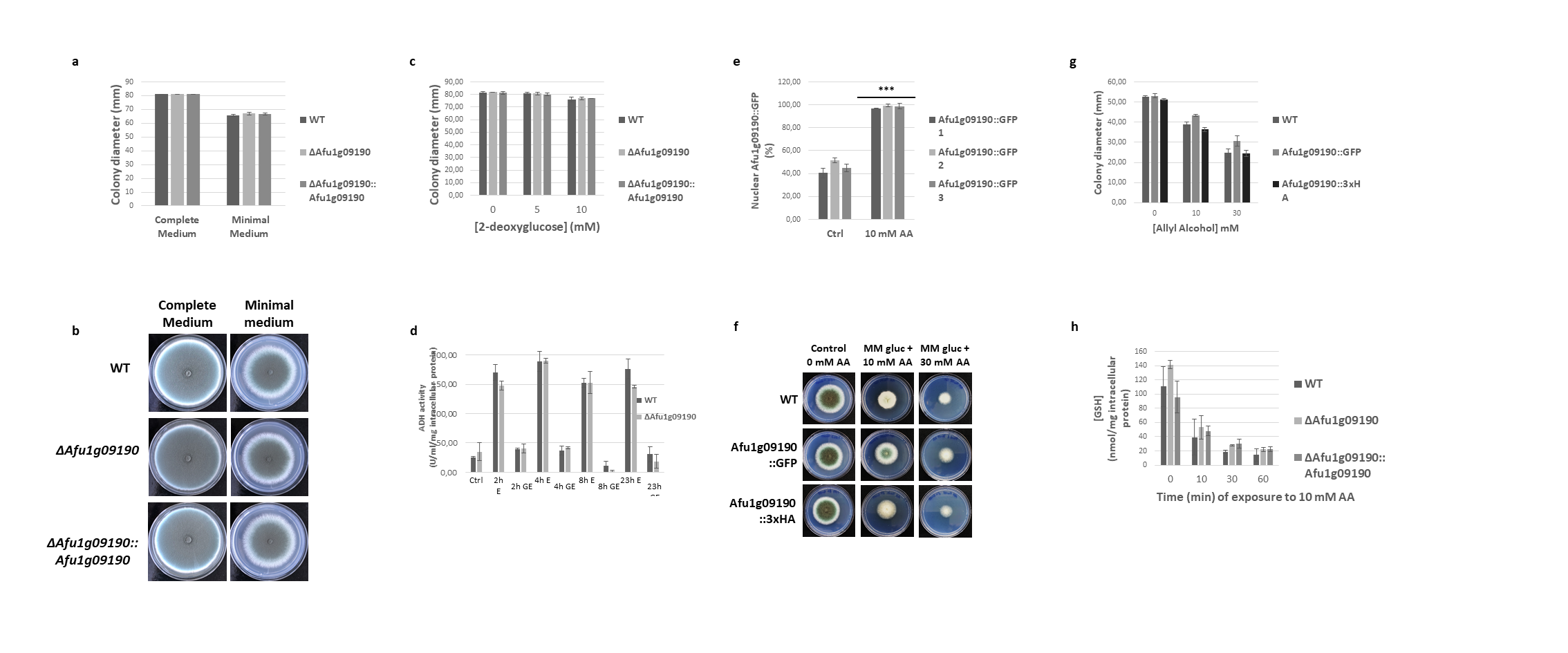

Supplement: S1 Fig — The ΔAfu1g09190 strain does not present any growth defects in non-stress conditions (A-,B) and in the presence of 2-deoxyglucose (2DG) (C). Strains (WT = wild-type) were grown for 5 days from 105 spores at 37°C on complete or glucose minimal medium (GMM) or on MM supplemented with xylose and increasing concentrations of 2DG, before colony diameter was measured. Standard deviations represent biological triplicates. D, Alcohol dehydrogenase (ADH) activity is not de-regulated in the ΔAfu1g09190 strain. Strains were grown for 24 h in fructose minimal medium (ctrl = control) before being transferred to ethanol (E)- or ethanol and glucose (GE)-rich minimal medium for different time points. Standard deviations represent biological triplicates. E, Afu1g09190 localises to the nucleus after the addition of allyl alcohol (AA). Three independent Afu1g09190::GFP candidates were grown for 16 h in GMM at 30°C before 10 mM AA was added for 15 min. Germinated hyphae were viewed under a fluorescence microscope before GFP fluorescence and DAPI-stained nuclei were counted in 50 germlings and the percentage of nuclear Afu1g09190::GFP was calculated. Standard deviations represent three biological replicates (***P-value < 0.0005 in a paired, equal variance t-test comparing the AA with the control condition). F-G, The Afu1g09190 GFP- and 3xHA-tagged strains are functional and do not present growth defects. Strains were grown for 5 days from 105 spores at 37°C on GMM supplemented with increasing concentrations of AA, before colony diameter was measured. Standard deviations represent biological triplicates. H, The ΔAfu1g09190 strain is not impaired in intracellular glutathione (GSH) levels. Strains were grown for 24 h in glucose minimal medium (time point 0, control) before 10 mM AA was added for different time points and GSH concentrations were determined. Standard deviations represent biological triplicates. (TIF) [file ppat.1008645.s001.TIF]

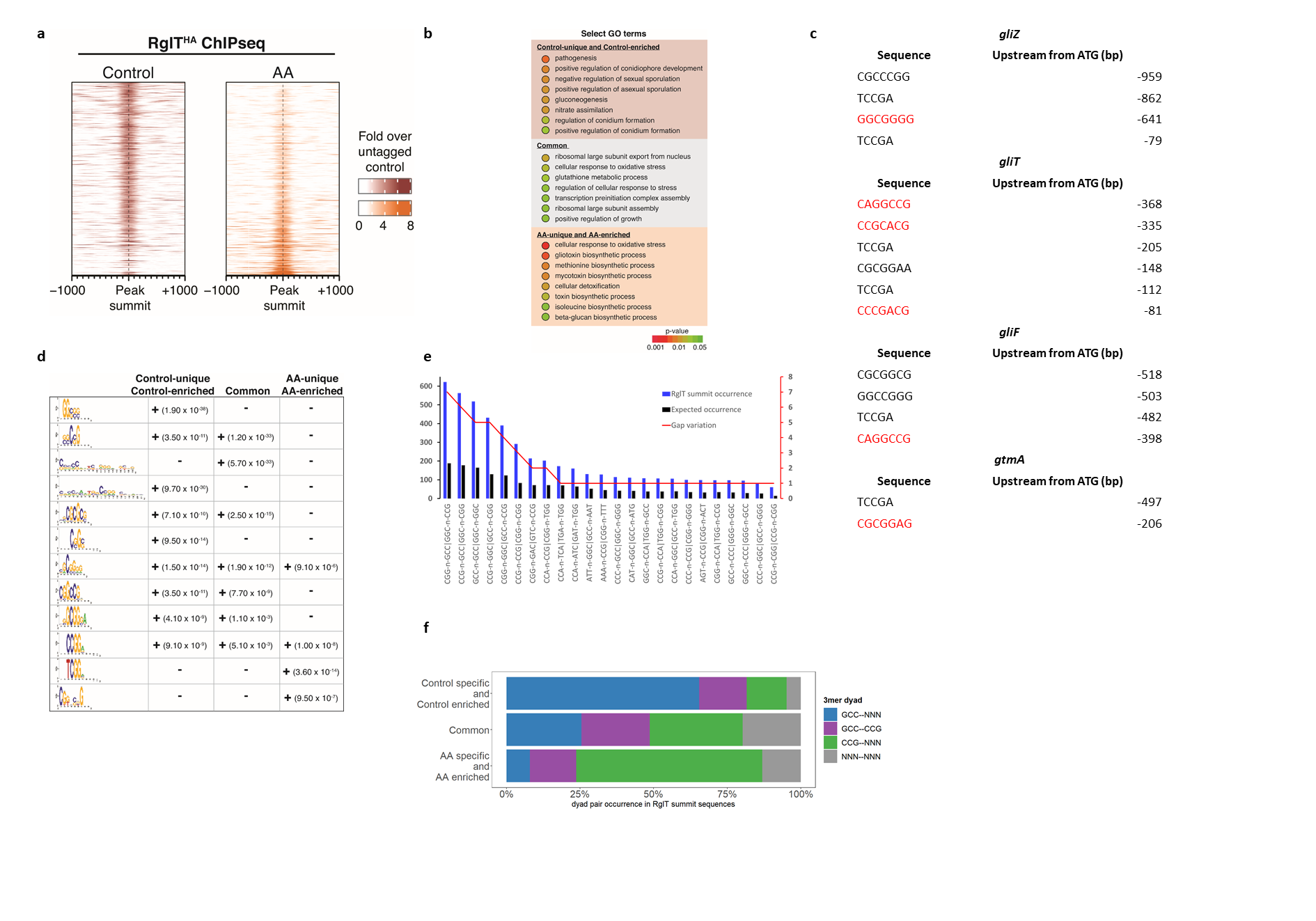

Supplement: S2 Fig — a. Genome-wide binding signals as determined by ChIP-seq (chromatin immunoprecipitation coupled to DNA sequencing) for the wild-type (WT) and RglT(Afu1g09190)::HA strains when grown for 24 h in glucose minimal medium (control = CTRL) or after the addition of 10 mM allyl alcohol (AA) for 30 min. b, Gene ontology (GO) analysis of all the genes that were significantly bound by RglT::HA, as determined by ChIP-seq. Binding site preferences were divided into 3 categories: i) sites unique to or enriched for binding in the control condition, ii) sites that were bound with equal strength in both the control and AA conditions and iii) sites unique to or enriched for binding in the AA condition. Also depicted are the p-values for each GO category. c, DNA sequence and localisation of putative RglT binding motifs, based on the ChIP-seq data, that were enriched in the presence of AA, in the promoter regions of gliT, gliF and gtmA. Highlighted in red are the sequences that were assayed by ChIP-qPCR. d, MEME (Multiple EM for Motif Elicitation)-ChIP analysis of the 500 bp region surrounding the peaks identified during ChIP-seq. Shown are the sequences of potential binding motifs together with the respective p-value for each of the 3 categories described in (a). A trace (-) signifies that the particular binding motif was not identified in the respective condition. e, 3mer dyad pair enrichment analysis of 200bp summit sequences from combined peaks. GC rich 3mer dyads are significantly enriched in RglT binding region. 3mer sequences CCG|CGG and GCC|GGC are found in most of the significantly enriched dyad pairs in combined RglT peak summit sequences. Some dyad pairs had multiple variants in terms of gap sequence length between two 3mers. These gap variations for each dyad pair are shown using secondary axis in the plot. f, AA enriched peaks and Control enriched peaks show preference for dyad pairs with specific 3mer sequence. Dyads with CCG|CGG 3mer occur more frequently in AA specific [file ppat.1008645.s002.TIF]

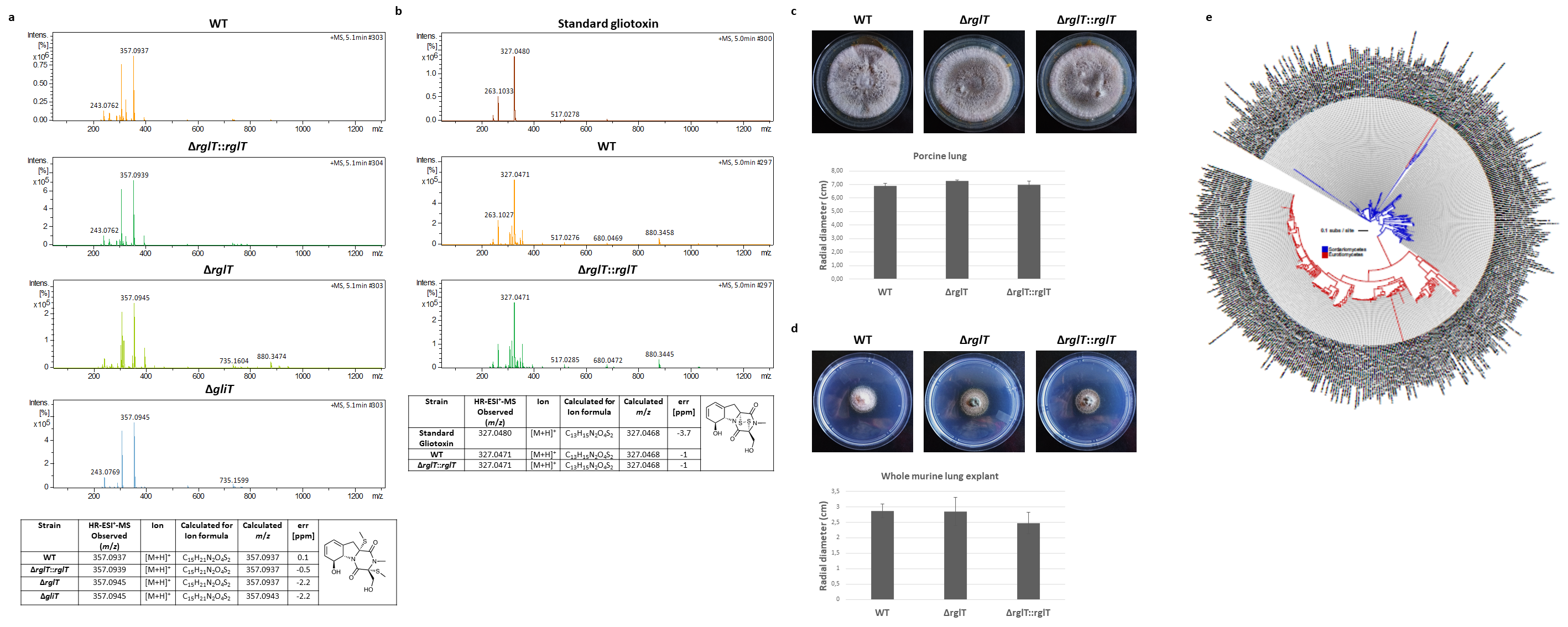

Supplement: S3 Fig — HRESIMS analysis for (a) bisdethiobis(methylthio)GT (BmGT) and for (b) GT in extracts of supernatants of strains that were grown for 72 h in GT-inducing conditions. Tables show the chemical structure, formula and mass-to-charge ratios (m/z) for both protonated GT and BmGT ([M+H+]). c-d, The ΔrglT strain has no growth defect in the presence of porcine lung (c) and whole murine lung explants (d). The upper panel shows pictures of growth after 5 days at 37°C from 105 spores of the wild-type, ΔrglT and ΔrglT::rglT strains on plates containing porcine lung or one murine lung. The lower panel depicts the graph of the radial growth from the upper panel. Standard deviations represent three biological replicates. (e) A three-gene phylogeny of 458 taxa in the fungal classes Eurotiomycetes and Sordariomycetes. Red branches refer to taxa from the Eurotiomycetes while blue branches refer to taxa from the Sordariomycetes. Branch lengths correspond to amino acid substitutions / site. (TIF) [file ppat.1008645.s003.TIF]

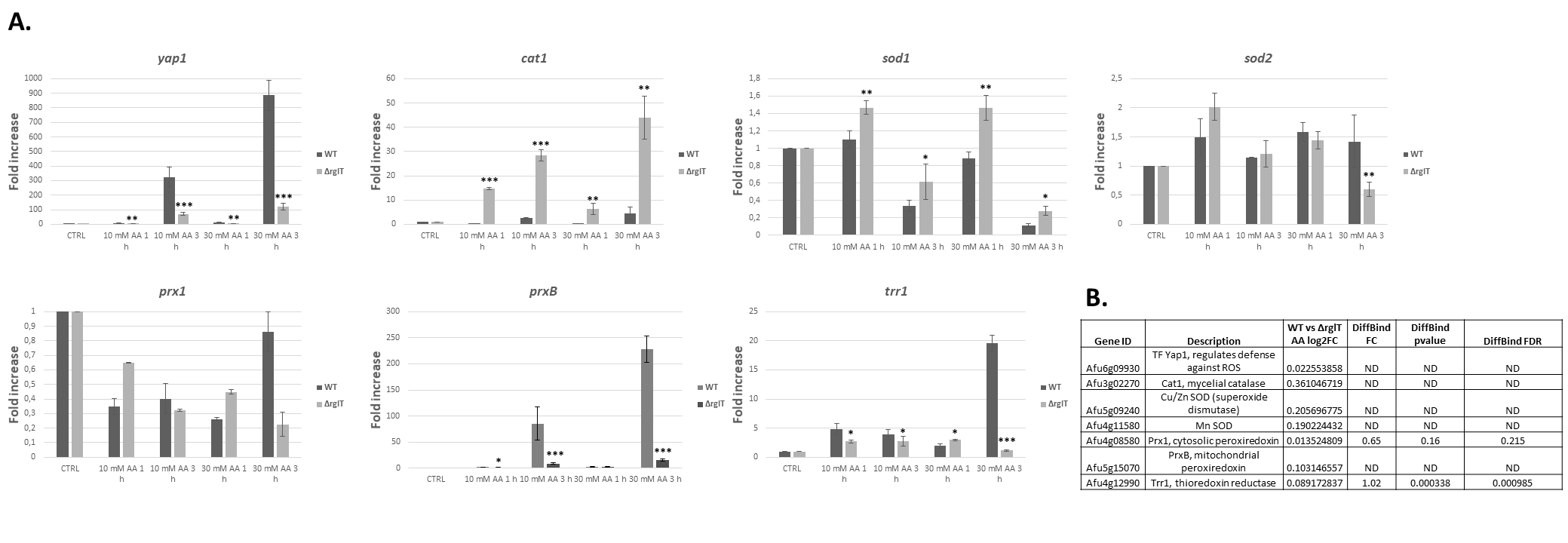

Supplement: S4 Fig — (A), Expression of genes, as determined by qRT-PCR when the WT and ΔrglT strains were incubated in the presence of 10 and 30 mM allyl alcohol (AA) for 1 h and 3 h. Gene expression is given as fold induction in comparison to the control (CTRL, glucose minimal medium), AA-free condition. Gene expression was normalized by actin. Standard deviations represent three biological replicates (*P-value < 0.01; **P-value < 0.001; ***P-value < 0.0001 in a two-way ANOVA test). (B) Table summarizing gene ID, gene annotation, log2 fold change (log2FC as determined by RNA-sequencing), and differential binding (Diffbind) FC, p-values and false discovery rates (FDRs) as determined by chromatin immunoprecipitation coupled to DNA sequencing (ChIP-seq) for the genes shown in (A). (TIF) [file ppat.1008645.s004.TIF]

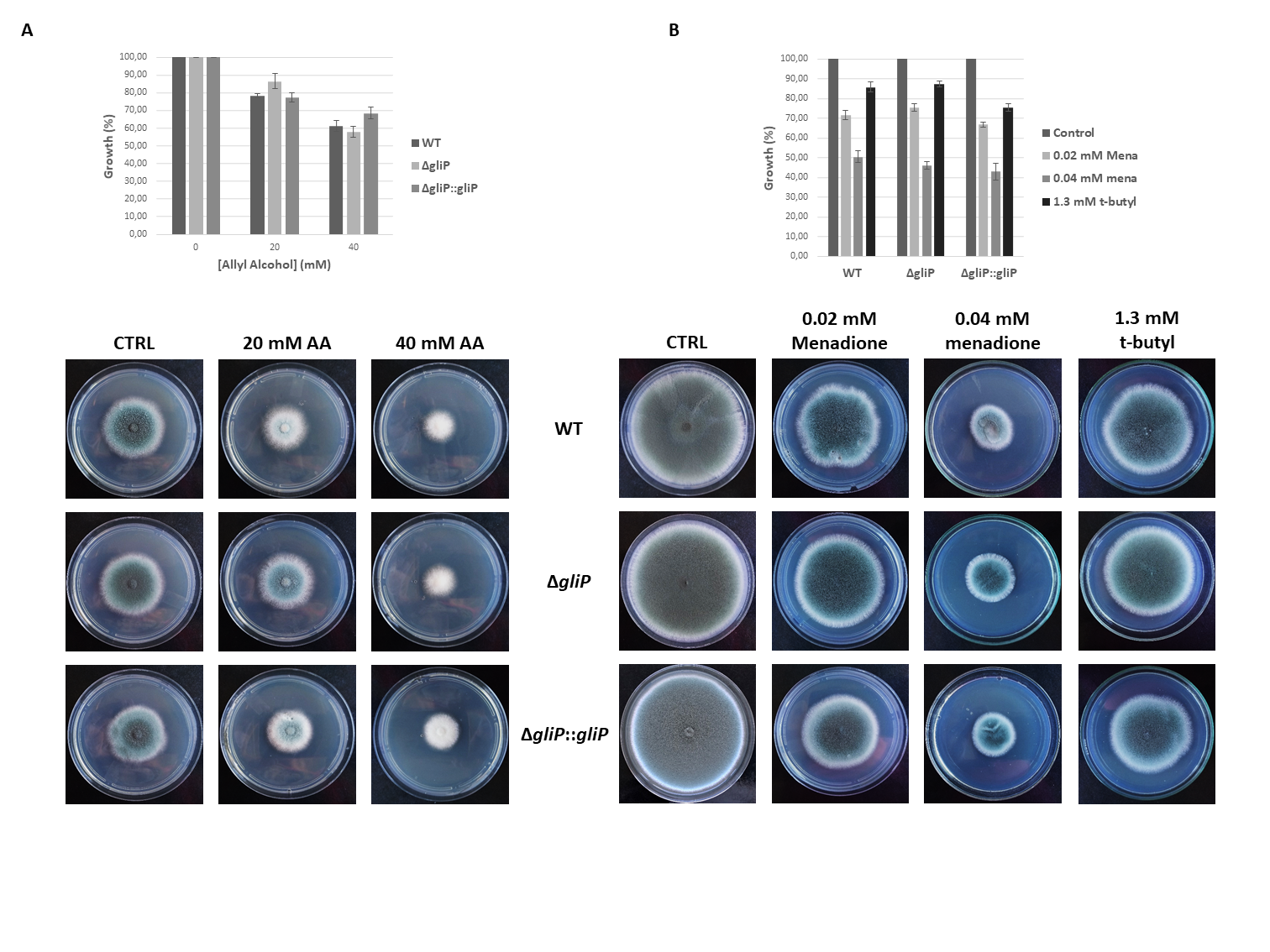

Supplement: S5 Fig — Strains were grown for 5 days from 105 spores at 30°C (allyl alcohol–AA) or 37°C on glucose minimal media supplemented with increasing concentrations of the oxidative stress-inducing compounds (A) AA and (B) menadione (mena) and t-butyl hydroperoxide (t-butyl). Graphs indicate the % of growth in the presence of the respective drug with respect to the control condition (without drug). Graphs are the quantitation of radial growth of the pictures shown in the same panel, with standard deviations representing three biological replicates. (TIF) [file ppat.1008645.s005.tif]
