# Supplementary material for: The Aspergillus fumigatus transcription factor RglT is important for gliotoxin biosynthesis and self-protection, and virulence
Source: PLoS Pathog. 2020 Jul 15;16(7):e1008645. doi: 10.1371/journal.ppat.1008645 (PMC7384679; doi:10.1371/journal.ppat.1008645)
Supplement: S1 Table — (DOCX) [file ppat.1008645.s011.docx]

**Supplementary Table 1**. Strains used in this study.

| **Name** | **Genotype** | **Reference** |
| --- | --- | --- |
| CEA17 | Wild-type, *MAT1-1* | [1] |
| Δ*rglT* | *MAT1-1*; *delta* *rglT::hph* | [2] |
| Δ*rglT*::*rglT* | *MAT1-1*; *delta rglT; delta rglT*::*rglT* | This study |
| RglT::HA | *MAT1-1*; *rglT::ha* | This study |
| Afu1g09190::GFP | *MAT1-1*; *Afu1g09190::gfp* | This study |
| TN02a3 | *pyrG89; argB2 delta-nkuA::argB; pyroA4; veA1* | [3] |
| ΔAN1368 | *delta AN1368::pyrG; argB2 delta-nkuA::argB; pyroA4; veA1* | This study |
| ΔAN1368::AN1368 | *delta AN1368; delta AN1368::AN1368; argB2 delta-nkuA::argB; veA1* | This study |
| ATCC26933 | Wild-type | [4] |
| Δ*gliT* | *delta gliT::ptrA* | [4] |
| Δ*gliT*::*gliT* | *delta gliT*; *delta gliT::gliT* | [4] |
| Δ*gliP* | *delta gliP::phleo* | [5] |
| Δ*gliP*::*gliP* | *delta gliP*; *delta gliP::gliP* | [5] |

1. Fedorova ND, Khaldi N, Joardar VS, Maiti R, Amedeo P, Anderson MJ, et al. Genomic islands in the pathogenic filamentous fungus Aspergillus fumigatus. PLoS Genet. 2008;4. doi:10.1371/journal.pgen.1000046
2. Furukawa T, van Rhijn N, Fraczek M, Gsaller F, Davies E, Carr P, et al. The negative cofactor 2 complex is a key regulator of drug resistance in Aspergillus fumigatus. Nat Commun. 2020;11. doi:10.1038/s41467-019-14191-1
3. Hoffmann B, Eckert SE, Krappmann S, Braus GH. Sexual diploids of Aspergillus nidulans do not form by random fusion of nuclei in the heterokaryon. Genetics. 2001;157: 141–147.
4. Schrettl M, Carberry S, Kavanagh K, Haas H, Jones GW, O’Brien J, et al. Self-protection against gliotoxin-a component of the gliotoxin biosynthetic cluster, gliT, completely protects Aspergillus fumigatus against exogenous gliotoxin. PLoS Pathog. 2010;6. doi:10.1371/journal.ppat.1000952
5. Kupfahl C, Heinekamp T, Geginat G, Ruppert T, Härtl A, Hof H, et al. Deletion of the gliP gene of Aspergillus fumigatus results in loss of gliotoxin production but has no effect on virulence of the fungus in a low-dose mouse infection model. Mol Microbiol. 2006;62: 292–302. doi:10.1111/j.1365-2958.2006.05373.x
