# Supplementary material for: The Aspergillus fumigatus transcription factor RglT is important for gliotoxin biosynthesis and self-protection, and virulence
Source: PLoS Pathog. 2020 Jul 15;16(7):e1008645. doi: 10.1371/journal.ppat.1008645 (PMC7384679; doi:10.1371/journal.ppat.1008645)
Supplement: Attachment — Submitted filename: Answers to the Reviewers.docx [file ppat.1008645.s012.docx]

**Answers to the Reviewers**

**Reviewer #1**: The authors report on the study of a novel putative transcriptional regulator (rglT) in A. fumimgatus. Originally identified in a screen for TFs involved in carbon catabolite repression, the rglT mutant was found to be important for (among other things) gliotoxin production and self-protection via transcriptional regulation of gli genes.  The authors provide excellent, in-depth data to support the conclusions surrounding the role of rglT in gliotoxin production and protection. The authors also provide an intriguing characterization of the presence and role of rglt homologs in non-GT producing organism, A. nidulans. The authors do also provide data supporting a role for rglT in immune modulation and virulence, showing that the mutant produces an enhanced inflammatory response. Overall, this is a strong manuscript. However, the in vivo links between rglT-mediated gliotoxin production and subsequent immune modulation need to be strengthened.  I have a few comments
for the authors to consider.

**Major comments:**

1. The conclusion that lack of gliotoxin production by the rglT deletion mutant is truly the causative factor of the increased immune response (and therefore decreased virulence) noted is difficult to fully support. Adding GT back to this system would inhibit rglT deletion-mutant growth. As the referenced study regarding the importance of GT in leukotriene production (9) employed a corticosteroid treated mouse and measured different immunological endpoints than the current study, it would be of great value to include the gliP deletion mutant into the data presented in Fig 4. This is crucial in determining that the outcomes here could be replicated with a GT-deficient strain and ensure that they are not from non-GT associated effects of rglT deletion. As it stands the data is certainly correlative, but it is not yet a conclusive link.

Answer: We have removed the immunocompetent mouse model from this study as histopathology showed severe inflammation throughout the lung tissue and little fungal dissemination. Instead, we have added survival curve, histopathology and fungal burden of a chemotherapeutic murine model of IPA for Δ*rglT* (lines 386 to 441, Figure 4). In addition, we provide data that shows that the avirulence of the Δ*rglT* strain is not due to cell wall changes or growth defects but may be associated to oxidative stress resistance defects among others. We have further elaborated on this in the discussion (lines 575-595) and suggest that the observed virulence phenotype may be related to defects in oxidative stress resistance and/or additional mechanisms that are essential for *in vivo* survival and that have not been characterised *in vitro* as of date. This remains subject to future studies. Due to the COVID-19 situation, we were not able to do virulence experiments with Δ*gliP* mutant. Since the Δ*gliP* strain also does not produce GT but is still virulent in a chemotherapeutic murine model of IPA (has been published), our results strongly suggest that the absence of GT in Δ*rglT* strain is not the major cause for the observed reduction in virulence in leukocyte-depleted mice.

2. The mortality levels associated with the WT and complemented strains are surprising for a C57Bl6 immunocompetent mouse model. Is dissemination occurring in this model? In the surviving mice infected with the rglT deletion mutant, is fungal burden cleared? It would be valuable to know if the model were carried out for a few more days, would these mice also succumb to infection?

Answer: We have removed the immunocompetent mouse model from this study as histopathology showed severe inflammation throughout the lung tissue and little fungal dissemination. Instead, we have added survival curve, histopathology and fungal burden of a chemotherapeutic murine model of IPA for delta rglT (lines 386 to 441, Figure 4).

**Minor issues:**

The authors utilize the term/title of  “Growth inhibition (%)” to label the y-axes of many graphs throughout the data. However, what they are really reporting is not a % growth inhibition, but a % of growth vs a non-treated control. For example, the first graph of Fig 5C surely does not report 100% growth inhibition at 0 ug/ml of gliotoxin. These graphs should be edited for clarity.

We have changed the y-axis label of all graphs showing radial growth (Figures 1, 3 and 5) from “Growth inhibition (%)” to “Growth (%)”. Furthermore, we have specified in the figure legends what this % of growth refers to.

**Reviewer #2**: The authors report the characterization of an Aspergillus TF, RglT, that regulates several members of the GT biosynthesis gene cluster. RglT-dependent genes include gliT that encodes an oxidoreductase involved in resistance to exogenous GT. The authors show that the RglT-deficient strain exhibits reduced virulence in an immune competent model of disease.

**Major comments:**

1. The authors' model imply that loss of gliotoxin production is linked to the in vivo virulence phenotype. However, this conjecture is not tested experimentally. It would be informative to determine gliotoxin levels from the lungs of infected mice, among the three groups tested. The authors' model would predict that exogenous addition of gliotoxin should thus restore virulence (in mice and/or in macrophages in vitro) in the RglT-deficient strain.

Answer: We have removed the immunocompetent mouse model from this study as histopathology showed severe inflammation throughout the lung tissue and little fungal dissemination. Instead, we have added survival curve, histopathology and fungal burden of a chemotherapeutic murine model of IPA for Δ*rglT* (lines 386 to 441, Figure 4). In addition, we provide data that shows that the avirulence of the Δ*rglT* strain is not due to cell wall changes or growth defects but may be associated to oxidative stress resistance defects among others. We have further elaborated on this in the discussion (lines 575-595) and suggest that the observed virulence phenotype may be related to defects in oxidative stress resistance and/or additional mechanisms that are essential for *in vivo* survival and that have not been characterised *in vitro* as of date. This remains subject to future studies. Due to the COVID-19 situation, we were not able to measure gliotoxin in murine lungs and do virulence experiments with Δ*gliP* mutant. Since the Δ*gliP* strain also does not produce GT but is still virulent in a chemotherapeutic murine model of IPA, our results strongly suggest that the absence of GT in Δ*rglT* strain is not the major cause for the observed reduction in virulence in leukocyte-depleted mice.

Another possible explanation for the in vivo phenotype relates to slow in vivo growth or to potential effects on carbon catabolite repression pathways. Does RglT regulate CreA in vitro or in vivo?

We have performed growth of the delta rglT strain in the presence of ground porcine lung and have not seen any significant differences with the wild-type and complemented strains, suggesting that this strain does not have problems growing in the presence of lung material. We have added pictures and bar graphs showing porcine lung growth as supplementary material (line 404 and thereafter, supplementary Fig 3c). Furthermore, our RNA-seq data showed that creA is not regulated by RglT and our ChIP-seq data did not find binding of RglT to the creA promoter region in vitro. Furthermore, our data show that no defects in carbon catabolite repression were observed (first section of results and supplementary fig 1), suggesting that RglT does not regulate CreA.

2. The mouse model of infection should include quantitative fungal growth and histopathology as endpoints. Are the enhanced inflammatory responses observed in the mice infected with the deletant mutant may be inversely proprotional to fungal growth? If so, this may be due to changes in the fungal cell wall or due to GT-dependent inhibition of innate immune responses. This should be addressed experimentally. Do macrophages display similar or different degrees of inflammation (e.g. cytokine production) when challenged with the three strains in live resting and heat-killed swollen states? Use of HK conidia would allow you to parse out the contribution of GT production versus changes in the cell wall to the readout. In summary the authors should attempt to provide additional insight into why the deletant strain is hypovirulent.

Answer: We have removed the immunocompetent mouse model from this study as histopathology showed severe inflammation throughout the lung tissue and little fungal dissemination. Instead, we have added survival curve, histopathology and fungal burden of a chemotherapeutic murine model of IPA for Δ*rglT* (lines 386 to 441, Figure 4). In addition, we provide data that shows that the avirulence of the Δ*rglT* strain is not due to cell wall changes (in vitro macrophage experiments with HK and LR conidia) or growth defects but may be associated to oxidative stress resistance defects among others. We have further elaborated on this in the discussion (lines 575-595) and suggest that the observed virulence phenotype may be related to defects in oxidative stress resistance and/or additional mechanisms that are essential for *in vivo* survival and that have not been characterised *in vitro* as of date. This remains subject to future studies. Due to the COVID-19 situation, we were not able to do virulence experiments with Δ*gliP* mutant. Since the Δ*gliP* strain also does not produce GT but is still virulent in a chemotherapeutic murine model of IPA, our results strongly suggest that the absence of GT in Δ*rglT* strain is not the major cause for the observed reduction in virulence in leukocyte-depleted mice.

3. Non-parametric tests should be used consistently for statistical comparisons. Use of the student-t-test is not appropriate for any panel in Fig. 1, 3, 4 and 5 (as an example) since three groups are being compared.

We have applied a two-way ANOVA test for multiple comparisons in figures 1,3 and 5. Statistical details were changed in each figure legend and, if required, in each figure. Student t-test remained in place for non-multiple comparisons such as in figure 3f (ChIP-qPCR) and in figure 4a,b (phagocytosis and viability assays) where one strain was compared with the other in a single condition.

**Minor issues:**

1. Introduction - please cite contemporary reviews, written in the past three years.

All review references have been replaced by reviews from 2016-2019.

2. 118 - MPO results in the production of the hypochlorous acid rather than hydrochloric acid.

Line 117: “hydrochloric” has been changed to “hypochlorous”.

3. 136 - GT blocks NF-kB - this should be included.

Line 131: We have added the following sentence: “Furthermore, GT inhibits the production of pro-inflammatory cytokines, such as NFkB, by macrophages^3^”.

4. 153 - I believe that the term "master" is overstated here. Please omit. Regulation is apparent under defined in vitro conditions (allyl alcohol) and may not be present under alternative GT-inducing conditions.

Line 153: We have omitted “master regulatory”.

5. 190 - Please do not use "data not shown" in the manuscript. Show the data, even if negative.

All mention of data, which was referred to as “data not shown”, was removed from the manuscript.

6. 202 - Please omit "in a first instance". Could state - In the first series of experiments, the transcriptional response....

Line 199: “in a first instance” was changed to “in the first series of analyses”.

7. 369-370 - Please delete and rephrase "through an unidentified mechanism that likely involves regulation of gliT and gtmA expression.

Line 374: Sentence was rephrased to “through an unidentified mechanism that likely involves regulation of gliT and gtmA expression”.

8. The labeling in Fig 2 should be more legible - please increase font size for panels 2b and 2c.

Font sizes in figure panels 2b and 2c have been increased.

**Reviewer #3**: In this report, Ries and colleagues describe novel transcription factor, RglT, that they initially identified from a screen for AA sensitive mutants.  By employing an impressive array of molecular analyses (including RNA-seq, ChIP-seq, gene deletion) the authors convincingly demonstrate that RglT regulates the gliotoxin biosynthetic cluster and that the gene is required for gliotoxin production.  This aspect of the story, in addition to its rigor, is furthermore well-presented and should be of keen interest to the A. fumigatus research community.  However, the authors employ an immunocompetent mouse model and conclude that GliT is important for virulence in that context, presumably due to the loss of gliotoxin production in vivo.  I note some issues with this model and the author’s conclusions, which I feel should be addressed prior to publication (“major” comments).  In addition, a variety of “minor” comments are provided that, if addressed, would improve the
presentation and/or clarity for the reader.  All comments can be found in the attached document.

**Major comments**:

My major comments are centered on the animal model and the central conclusions drawn from those experiments. Two points are noted:

1) I am quite surprised to see that a single inoculum in an immunocompetent mouse results in such high mortality rates. As far as I can tell, such a model, at least with this *A. fumigatus* background and dosage, has not been previously published. I am not questioning the results, but it seems important that the authors provide additional pathological information to supplement the cumulative mortality data. For example, they conclude that the *rglT* mutant is hypovirulent due to enhanced neutrophil recruitment and fungal clearance. However, I would be surprised to see much fungal growth in even the WT-infected animals, and would rather assume that death is due to an inflammatory response. In this context then, I would actually predict increased mortality in the mutant! Therefore, I think histology at both early (24h) and later (past 72h) timepoints would provide important insight in to the amount of fungal growth and inflammation in WT v ∆*rglT* strains and supplement the authors flow cytometry data. Even fungal burden assessment alone (CFU or qPCR) would provide some support of their claim that the mutant is cleared more quickly.

Answer: We have removed the immunocompetent mouse model from this study as histopathology showed severe inflammation throughout the lung tissue and little fungal dissemination. Instead, we have added survival curve, histopathology and fungal burden of a chemotherapeutic murine model of IPA for Δ*rglT* (lines 386 to 441, Figure 4). In addition, we provide data that shows that the avirulence of the Δ*rglT* strain is not due to cell wall changes or growth defects but may be associated to oxidative stress resistance defects among others. We have further elaborated on this in the discussion (lines 575-595) and suggest that the observed virulence phenotype may be related to defects in oxidative stress resistance and/or additional mechanisms that are essential for *in vivo* survival and that have not been characterised *in vitro* as of date. This remains subject to future studies. Due to the COVID-19 situation, we were not able to do virulence experiments with Δ*gliP* mutant. Since the Δ*gliP* strain also does not produce GT but is still virulent in a chemotherapeutic murine model of IPA, our results strongly suggest that the absence of GT in Δ*rglT* strain is not the major cause for the observed reduction in virulence in leukocyte-depleted mice.

2) The authors suggest that the virulence defect of the ∆*rglT* is due to the loss of gliotoxin production and the consequent accumulation of leukotriene in the tissue. However, as RglT is a transcription factor, it could be regulating numerous other GT-independent phenotypes (e.g. oxidative stress sensitivity) that could contribute to its hypovirulence. Therefore, their conclusion should be supported by the inclusion of a gliotoxin-deficient mutant other than GliT. A *gliP* mutant would be a good choice and I don’t think it has been run in an immunocompetent model.

Answer: Please, see previous answer.

3) Even if gliotoxin does play a role in virulence in this murine model, immunocompetent individuals are not typically susceptible to infection. Therefore, the authors should consider testing their mutant in a more relevant, i.e. immunosuppressed, model of disease. In this way, the author’s ∆*rglT* data could be directly compared to previously published data concerning the virulence of gliotoxin mutants.

Answer: We have added survival curve, histopathology and fungal burden of a chemotherapeutic murine model of IPA for Δ*rglT* (lines 386 to 441, Figure 4). In addition, we provide data that shows that the avirulence of the Δ*rglT* strain is not due to cell wall changes or growth defects but may be associated to oxidative stress resistance defects among others. We have further elaborated on this in the discussion (lines 575-595) and suggest that the observed virulence phenotype may be related to defects in oxidative stress resistance and/or additional mechanisms that are essential for *in vivo* survival and that have not been characterised *in vitro* as of date. This remains subject to future studies. Due to the COVID-19 situation, we were not able to do virulence experiments with Δ*gliP* mutant. Since the Δ*gliP* strain also does not produce GT but is still virulent in a chemotherapeutic murine model of IPA, our results strongly suggest that the absence of GT in Δ*rglT* strain is not the major cause for the observed reduction in virulence in leukocyte-depleted mice.

**Minor Comments**

Figure 1: It appears that the bar graphs are a quantitation of the radial growth plates shown below. Is that right? If so, that should be explicitly stated. Also, if that is true, than the graphs and the plates are the same experiment and could be grouped together in the subpanels, e.g. panel ‘a’ is allyl alcohol, panel ‘b’ is acrolein, etc.

Graphs and plate pictures of figure 1 have been grouped together into panels and headings have been added. Furthermore, the legend title was changed to: “Graphs indicate the % of growth in the presence of the respective drug with respect to the control condition (without drug). Graphs are the quantitation of radial growth of the pictures shown in the same panel, with standard deviations representing three biological replicates (*P-value < 0.05; **P-value < 0.005; ***P-value < 0.0005 in a paired, one-tailed t-test).”

Line 174: *“…but presented no growth defects in non-stress conditions (Supplementary Fig. 1a-b) as well as in the presence of 2- deoxyglucose (2DG)…”*

Change “as well as” to “nor”.

Line 172: “as well as” was changed to “nor”.

Line 230: *“We then further focused on genes that were significantly differently regulated between both strains in the presence of AA…”*

The wording seems strange. Perhaps change to “differentially regulated in the mutant” or differentially regulated between the strains”.

Line 227: Sentence was rephrased to: “focused on genes that were significantly differentially regulated in the mutant strain, when compared to the WT strain, in the presence of AA”.

Line 242: *“… whereas the ΔAfu1g09190 strain (35 μg/ml) was significantly (p-value < 0.05) more sensitive to GT when compared to the WT (70 μg/ml) strain.”*

The authors should refer to a particular figure here.

Line 241: The readers are referred to Table 1, which was added to the manuscript. We also added the MIC methodology (lines 664-669).

Line 267: *“A total of 538 sites were bound by Afu1g09190 genome-wide under the two conditions. Among these, 160 sites were constitutively bound by Afu1g09190::3xHA (i.e. common targets) with the remaining 272 and 106 binding events being unique to or enriched in the control and AA conditions, respectively (Fig. 2d; Supplementary File 3).”*

Perhaps the authors could comment on the number/proportion of genes in which RglT was bound to the predicted promoter region of a gene, as opposed to some other intragenic or intergenic region. I didn’t see this mentioned in the Methods or elsewhere in the manuscript.

Line 268. We have added the following sentences to address the question on the number/proportion of predicted promoter gene regions bound by RglT: “Systematic assignment of intragenic and intergenic binding sites to nearest genes revealed that the Afu1g09190 intragenic binding sites were primarily located at the 5’ UTR (untranslated) region of the genes (219 sites) as opposed to very few binding sites at 3’ UTR (35 sites) regions and within exons (18). On the other hand, 130 intergenic binding sites were within promoter region [-500 bp to TSS (transcription start site)] and the remaining 139 intergenic sites were further upstream than 500 bp from the TSS. 15 Afu1g09190 binding sites were not associated with any gene as they were at convergent gene intergenic regions. Altogether, 481 unique genes were annotated with Afu1g09190 peaks.” Furthermore, we have also added the following sentence to the materials and methods section (line 696): “RglT binding sites were assigned to nearest genes in *A. fumigatus* reference annotation using inhouse script.”

Line 306: *“…through performing HPLC on culture supernatants of the WT, ΔrglT and ΔrglT::rglT strains, when grown in GT-producing conditions.”*

The authors should explicitly state what this GT-producing condition is at this point of the text (i.e. Czapek-Dox) along with an appropriate reference. The authors might also clarify why this medium was used since AA, used up to this point, appears to be a GT-producing condition!

Line 311: The following sentence was added: “Strains were grown in Czapek-Dox broth for 72 h as this medium has previously been shown to result in detectable gliotoxin levels in culture supernatants^12^”.

Line 377: *“The capacity of bone marrow-derived macrophages (BMDMs) to kill wild-type (WT), ΔrglT and ΔrglT::rglT conidia was determined and the ΔrglT strain was found to be significantly more susceptible (P-value < 0.05) to macrophage-mediated killing (Fig. 4a).”*

Upon reviewing the methods section for this experiment, it seems that an alternative explanation for the results is that the ∆*rglT* is not phagocytosed as efficiently as the wild-type, i.e. there are fewer conidia within the macrophages. Are there any data concerning this?

We have added data to figure 4a which shows that the delta rglT strain is phagocytized significantly more than the WT and complemented strains. This is therefore in agreement with the killing assay, showing that a higher % of conidia are phagocytised and subsequently killed by the macrophages.

Line 381: “ The capacity of bone marrow-derived macrophages (BMDMs) to phagocytize and kill wild-type (WT), Δ*rglT* and Δ*rglT*::*rglT* conidia was determined and the Δ*rglT* strain was found to be significantly more susceptible (P-value < 0.05) to macrophage-mediated phagocytosis and killing (Fig. 4a).”.

Line 477: *“These results confirm that RglT regulates the expression of GT self-protection genes when GT is added exogenously to cultures of both A. fumigatus and A. nidulans and suggest a conserved function of RglT for self -protection, even in GT non-producing Aspergillus species.”*

In general, I am confused about the term “self-protection”. This term perhaps makes sense for organisms that produce gliotoxin endogenously, such as *A. fumigatus*. However, for organisms that do not produce GT, like *A. nidulans*, it wouldn’t be “self” protection, it would just be GT ‘resistance’. In any case, GT is provided exogenously in these experiments, so it can’t be concluded that RglT infers ‘self-protection’, strictly speaking.

Line 487: The sentence was changed to: “These results confirm that RglT regulates the expression of GT self-protection and resistance genes when GT is added exogenously to cultures of both *A. fumigatus* and *A.nidulans*, and suggest a conserved function of RglT for resistance against exogenous GT, even in GT non-producing *Aspergillus* species”.

.

Line 519: *“Furthermore, we also observed a transcriptional down-regulation of several genes of the fumagillin biosynthetic gene cluster (BCG).”*

Change ‘BCG’ to BGC.

Line 532: “BCG” was changed to “BGC”.

Line 567: *“This is in agreement with^9^, who showed…”*

This should read “This is in agreement with ref. 9” or, preferably, “This is in agreement with Konig et al.”

This sentence is no longer present in the manuscript due to removal of the immunocompetent murine model virulence data.

Supplementary Table 1: Furukawa is listed as a reference for the ∆*rglT* mutant; however, this citation is not listed in that supplementary document or the primary manuscript. Please add this.

Ref. #7: The full title is not included.

The reference for the ∆*rglT* mutant has been included. The full title for reference #7 has been included.
