# Supplementary material for: The Aspergillus fumigatus transcription factor RglT is important for gliotoxin biosynthesis and self-protection, and virulence
Source: PLoS Pathog. 2020 Jul 15;16(7):e1008645. doi: 10.1371/journal.ppat.1008645 (PMC7384679; doi:10.1371/journal.ppat.1008645)
Supplement: Attachment — Submitted filename: Answers to the Reviewers.docx [file ppat.1008645.s013.docx]

**Answers to the Reviewers**

Reviewer #1:

1) This revised ms examines the contribution of a novel transcription factor, RglT, to the biology & virulence of AF. A deletion mutant grew normally on standard media, but was growth impaired on allyl alcohol and agents that induce oxidative stress. An extensive transcriptome and chromatin ip analysis suggested that the TF regulates genes involved in the gliotoxin biosynthesis, which was confirmed by HPLC and LC-MS. Resistance to GT was also abolished in this mutant, indicating loss of self-protection, implicating gliT. Deletion of the homolog in A. nidulans, which does not producer GT, caused similar phenotypic effect with respect to GT, allyl alcohol, and oxidative stress. A phylogenetic analysis suggested that GliT-based protection mechanisms evolved before RglT-mediated regulation of GliT.

Overall, this is an excellent body of work that delves into the function of a novel transcription factor. It is an impressive body of work that provides important information that will be of interest to the fungal community. The data is novel and the ms is clearly written.

Answer: We thank the reviewer for the comments.

2) The 80% survival of the mice in this study, and very low fungal burden, is very impressive given the normal growth on minimal lab medium and ground porcine lung extracts (line 629). This is interpreted as the inability of the mutant to proliferate in lung tissue (line 434). Ground lung is different from lung tissue because it contains reduced substrates due to degradation of polymers. Can this strain grow effectively on a polymeric substrate like collagen or a whole lung explants in vitro? This would help separate any effects of residual host immunity in the lung from a failure to use complex substrates.

Answer: We have grown the WT, delta rglT and complemented rglT strains in the presence of whole murine lung explants *in vitro* for 5 days at 37C. These results show that there is no difference in growth between the strains in the presence of whole murine lungs, suggesting that the virulence defect is not due to poor growth in murine lungs. Results have been added as supplementary figure 3d and referred to in the manuscript (Page 15, lines 432 to 436): “In addition, growth of the Δ*rglT* strain on agar plates supplemented with ground porcine lung fragments and whole murine lung explants did not significantly differ from growth of the WT and Δ*rglT::rglT^+^* strains (Supplementary Fig. 3c-d), suggesting that the Δ*rglT* strain grows normally on mammalian lung tissue *in vitro*.”

We have also added this section to materials and methods (Page 22, lines 667 to 668): “Lungs were extracted from healthy C57BL/6 mice and one lung was transferred to a 1% w/v agarose plate.”

3) Some mutant conidia have increased loss of viability, either when they are harvested or after storage in the fridge. I may have missed this in the ms, but given the strong impact on virulence/burden, it is crucial to plate the conidial stocks used for inoculation to demonstrate no loss in viability. This is likely to have been done, but it should be stated.

Answer: This has indeed been done and the information has been added to the methodology section. In addition, we have added that spore suspensions used for in vivo infections were prepared freshly a day prior to murine infection (Page 28, lines 865 to 869): “*A*. *fumigatus* conidia suspensions were prepared freshly a day prior to infection, washed three times with PBS, counted and diluted to a concentration of 5.0 x 10^6^ conidia/ ml. The viability of the administered conidia was determined by growing them in serial dilutions on YAG medium, at 37°C.”

4) I did not see how many times the strong virulence defect was repeated. There should be at least one biological replicate.

Answer: The murine survival curves were carried out twice as now stated in the methodology section (Page 28, lines 871 to 872): “Survival curves were carried out in two independent experiments”.

Reviewer #2

1) In my review of the authors’ first submission, I felt that while the in vitro/molecular work was impressive and convincing, there were major concerns about the immunocompetent mouse model. A more conventional chemotherapy model is employed in this current submission and the result is the same i.e. the ∆rglT is attenuated for virulence. This is interesting because it has been demonstrated that gliotoxin mutants (i.e. ∆gliP) are not hypovirulent in a comparable, i.e. neutropenic, model; thus, and as the authors rightly note, the virulence phenotype ∆rglT is likely not related to its loss of gliotoxin production. While this in principle makes the ∆rglT even more intriguing, it is also a dual edge sword because there is no clear sense of why the mutant is, in fact, hypovirulent. The authors suggest that that the oxidative stress phenotype could be part of the explanation, but this remains unsubstantiated. First, and to the best of my knowledge, the link between oxidative stress sensitivity and virulence in A. fumigatus is generally not well-established. For example, the yap1 mutant, which is a global regulator of the A. fumigatus oxidative stress response (and regulated by rglT in this report), is not hypovirulent in a neutropenic model (Lessing et al., Eukaryot Cell). Second, it has already been demonstrated that the loss of gliotoxin production in A. fumigatus leads to a hypersensitivity to oxidative stress (gliK mutant). This may have not been tested for the gliP mutant of A. fumigatus, but a gliP mutant of Trichoderma virens is indeed hypersensitive to ROS (Vargas et al., 2014, Microbiology). So, we are left with the likely scenario in which the A. fumigatus gliP mutant has an oxidative stress phenotype with no virulence phenotype, at least in the this infection model.

All of the above is to say that while the manuscript provides rigorous and novel insights into gliotoxin regulation in A. fumigatus, it does not seem to provide novel insight into A. fumigatus pathogenesis. It may be difficult to elucidate the mechanism of the ∆rglT virulence defect since, as the authors mention, it is likely through a myriad of processes. One small thing the authors could do in this regard is pin down the relationship between other gliotoxin mutants and oxidative stress sensitivity. For example, if they were to demonstrate that the gliP mutant was not hypersensitive to ROS, this would strengthen the argument that the ∆rglT virulence phenotype is ROS-related.

Answer: We thank the reviewer for the comments. We have grown the WT, delta gliP and complemented gliP strains on plates containing the oxidative stress-inducing compounds allyl alcohol, menadione and t-butyl and found that no differences in growth between these strains exist. Data was described in Pages 15 and 16 (lines 459 to 460 and 461 to 465, respectively) and added as supplementary Fig 5: “In addition, we grew the WT, Δ*gliP* and Δ*gliP*::*gliP* strains in the presence of oxidative stress-inducing compounds in order to further determine whether the virulence defect of the Δ*rglT* strain is due to defects in oxidative stress resistance. A relationship between gliotoxin and oxidative stress has previously been noted [11,27,28], although the mechanism has not been described to date. Growth of the Δ*gliP* strain was not significantly different from the WT and Δ*gliP* complemented strains in these conditions (Supplementary Fig. 5).”

Furthermore, we have discussed the discrepancy between RglT regulating Yap1, whose deletion does not cause any virulence defects (Page 21, lines 628 to 636): “Yap1 has been described as the major regulator of the *A. fumigatus* response against oxidative stress [26,35], although it is not required for virulence in a chemotherapeutic murine model of IPA [35]. This discrepancy may be explained by the fact that RglT regulates many additional genes required for oxidative stress resistance, such as a variety of oxidoreductases that have not been characterized to date. In addition, differences in regulatory mechanisms of TFs are bound to exist between the laboratory in vitro and murine in vivo conditions as has previously been shown for TFs in *A. fumigatus* [36] and *Candida albicans* [37].”

2) The authors continually refer to the rglT mutant as “avirulent”, but this seems to be inaccurate since some animals in the infection group do die. The terms “hypovirulent” or “severely attenuated for virulence” would be more appropriate.

Answer: We have replaced the term “avirulent” with “hypovirulent” throughout the manuscript.

3) The authors demonstrate that the rglT mutant conidia are more readily phagocytosed and killed by macrophages, and this corresponds with an increased pro-inflammatory (cytokine) response by macrophages. They further conclude that this phenotype is not due to cell wall abnormalities in the mutant because swollen, heat-killed conidia are not hyperinflammatory (Figure 4C). I wonder, could the mutant be germinating faster, i.e. exposing beta-glucan faster, thus leading to increased uptake and killing sensitivity relative tot he wild-type? The colonial growth of the mutant seems normal enough, but perhaps there is a subtle difference in germination kinetics. In that regard, the authors seem not to mention the incubation time ahead of cytokine analysis. Was this also 4 hrs? In any case, this could be more clearly stated. Sorry, if I’m missing something.

Answer: Cytokine production by macrophages incubated with *A. fumigatus* conidia was also performed after 4 h at 37C in 0.5% CO_2_. We have added this information (Page 27, lines 840 to 842): “Cytokine production of macrophages incubated with conidia (1:5 macrophage/conidia ratio) for 4 h at 37°C in 5% CO_2_ was measured as described previously [56].”

4) Reference 7 in the Bibliography is still missing the full title!

Answer: Reference 7 was revised and the full title was added.

Reviewer #3

1) In this report by Ries, at al the authors identify RgIT as a novel transcription factor that regulates the production of the secondary metabolite gliotoxin in Aspergillus fumigatus. The authors employ a comprehensive array of technical approaches including RNA-seq, CHIP-seq, LC-MS and multiple gene-deficient Af strains to support the conclusion that RgIT is an important regulator of the gliotoxin biosynthetic cluster. Their data further suggest that RgIT regulates gliotoxin self-protection and oxidative stress resistance. The authors have edited the paper based on previous feedback and now present a more focused and well supported studies. Primary concerns and suggestions still revolve around the in vivo studies.

The authors test the impact of RgIT to Af virulence in a model of chemotherapy-induced immunosuppression. The survival curve of treated mice supports the conclusion that RgIT deficient strain is avirulent in vivo. What remains unclear is the mechanism behind the attenuation. The ex vivo cultures with BM-Macrophages suggest that immune cells are more efficient at phagocytosing and killing the RgIT mutant. Since the in vivo study is done with drugs that impair immune function it is unclear that in vivo, innate cells are similarly able to uptake and kill the mutant better. The authors suggest that the mutant is more susceptible to oxidative stress, likely to be mediated by the activity of NADPH oxidase in vivo but there is no experimental approach done to test this possibility

Answer: We thank the reviewer for the comments.

2) The data shown in Fig 4D and 4E appear to indicate rapid clearance of the mutant. By day 3 there seems to be undetectable amounts of fungal burden by PCR (Fig 4D) and barely any visible conidia (4E). Including a naïve control for reference to baseline would make the data on these panels more clear by facilitating the distinction between low burden and clearance. It would also be informative to perform a kinetic analysis starting at early time points before day

Answer: We have performed fungal burden of the PBS negative control mice and added this to Fig4D. Results show no statistical difference between PBS and fungal burden, suggesting that the delta rglT strain is cleared within the lungs (Page 14, lines 409 to 413): “Fungal burden in murine lungs showed a significant reduction of Δ*rglT* strain presence/growth in comparison with the WT and Δ*rglT::rglT^+^* strains that was not significantly different from the negative PBS control (Fig. 4d), suggesting that the Δ*rglT* strain was cleared from the lung tissue.”

We thank the suggestion of the reviewer, however based on the current results we do not believe it is necessary to perform a fungal burden kinetic analysis at earlier time points as our results strongly support clearance of the delta rglT strain from murine lungs.

3) The authors employ an ex vivo culture with ground porcine lung material to argue that the mutant does not have growth defects in the presence of lung material. The experimental approach does not directly rule out in vivo growth defects in the murine lung.

Answer: We have grown the WT, delta rglT and complemented rglT strains in the presence of whole murine lung explants *in vitro* for 5 days at 37C. These results show that there is no difference in growth between the strains in the presence of whole murine lungs, suggesting that the virulence defect is not due to poor growth in murine lungs. Results have been added as supplementary figure 3d and referred to in the manuscript (Page 15, lines 432 to 436): “In addition, growth of the Δ*rglT* strain on agar plates supplemented with ground porcine lung fragments and whole murine lung explants did not significantly differ from growth of the WT and Δ*rglT::rglT^+^* strains (Supplementary Fig. 3c-d), suggesting that the Δ*rglT* strain grows normally on mammalian lung tissue *in vitro*.”

We have also added this section to materials and methods (Page 22, lines 667 to 668): “Lungs were extracted from healthy C57BL/6 mice and one lung was transferred to a 1% w/v agarose plate.”

4) The authors suggest that the lower virulence of the RgIT mutant might be linked to enhanced sensitivity to oxidative stress. Have the investigators examined whether the RgIT mutant can grow better in BM-macrophages from NADPH-deficient mice?

Answer: We have performed phagocytosis and killing assays of BM-macrophages derived from CGD (chronic granulomatous disease) mice that have mutations in the NADPH (gp91^phox^ knockout CGD mice). Results show that CGD-derived macrophages are still able to significantly kill a higher number of delta rglT conidia suggesting that the virulence defect of this strain is not due to defects in oxidative stress resistance (Page 16, lines 466 to 475, figure 4a and b): “To definitely determine whether the Δ*rglT* strain has defects in oxidative stress resistance, phagocytosis and killing by macrophages derived from a murine model of CGD (chronic granulomatous disease) with NADPH deficiency was performed. CGD macrophages are still able to phagocytize (Fig. 4A) and kill (Fig. 4B) a significantly higher number of Δ*rglT* conidia, suggesting that the virulence defect of the Δ*rglT* strain is not due to defects in resistance to oxidative stress exerted by macrophages. Together, these results suggest that the inability of the Δ*rglT* strain to infect chemotherapeutic mice is due to unidentified mechanisms that are essential for *in vivo* survival and that have not been described *in vitro* as of date.”

We have also added a part in the discussion that discusses the results in context of these results and experiments suggested by the other reviewers (Pages 21 and 22, lines 616 to 625 and 626-to 636): “We therefore hypothesized that the Δ*rglT* strain hypovirulence may be related to defects in oxidative stress resistance. In favor of this theory is the observation that the Δ*gliP* strain is not sensitive to the here tested oxidative stress-inducing compounds. GT is known to have immunomodulatory functions[7–9] but it also induces oxidative stress in mammalian cell lines and interferes with GSH levels that can exacerbate the effects of GT[11]. Enzymes encoded by the *gli* genes appear to play different roles in oxidative stress protection as observed for *gliP* and *gliK* [27]. The relationship between GT and oxidative stress is therefore extremely complex and remains subject to future investigations. The Δ*rglT* strain is sensitive to GT and oxidative stress-inducing compounds, phenotypes that can be partially explained by RglT controlling the expression of genes encoding enzymes, such as Yap1, required for oxidative stress resistance. Yap1 has been described as the major regulator of the *A. fumigatus* response against oxidative stress [26,35], although it is not required for virulence in a chemotherapeutic murine model of IPA [35]. This discrepancy may be explained by the fact that RglT regulates many additional genes required for oxidative stress resistance, such as a variety of oxidoreductases that have not been characterized to date. In addition, differences in regulatory mechanisms of TFs are bound to exist between the laboratory *in vitro* and murine *in vivo* conditions as has previously been shown for TFs in *A. fumigatus* [36] and *Candida albicans* [37].”

5) In lines 134-135 the authors state: “Furthermore, GT inhibits the production of pro-inflammatory cytokines, such as NFkB, by macrophages[3]” . NFkB is not a cytokine, please revise.

Answer: This sentence has been changed to (Page 6, lines 134 to 136): “Furthermore, GT inhibits the production of pro-inflammatory cytokines secreted by macrophages and the activation of the NFkB regulatory complex”.

6) Throughout the paper the authors refer to the murine model as a “chemotherapeutic murine model of invasive aspergillosis” without giving any details in methods or figure legend exactly what drug treatment did they use. Please add that a combination of cyclophosphamide and hydrocortisone was used. While citing previous work for experimental detail is certainly appropriate, each paper needs sufficient detail for the reader to understand the experimental approaches used without having to read another paper.

Answer: We have added a brief description (including that cyclophosphamide and hydrocortisone was used for immunosuppression) of the methodology that was used to do the in vivo virulence assays, fungal burden and histopathology (Page 28, lines 862 to 872): “Briefly, mice were immunosuppressed intraperitoneally with cyclophosphamide on days -4, -1, and 2 prior to and post infection, and subcutaneously with hydrocortisonacetate on day -3 prior to infection. *A*. *fumigatus* conidia suspensions were prepared freshly a day prior to infection, washed three times with PBS, counted and diluted to a concentration of 5.0 x 10^6^ conidia/ ml. The viability of the administered conidia was determined by growing them in serial dilutions on YAG medium, at 37°C. Mice (n = 10/*A. fumigatus* strain) were infected by intranasal instillation of 1.0 x 10^5^ conidia diluted in 20 μl of PBS. Mice (n = 5) which received 20 μl of PBS served as the negative control. Survival curves were carried out in two independent experiments.”

In addition, we have added to the legend of figure 4 that mice were immunosuppressed with cyclophosphamide and hydrocortisone.

7) In Figure 4B the axis is labeled as %Lethality, it would be more appropriate to label as %conidia killed.

Answer: The y-axis of Figure 4B was re-labelled to “Killed conidia (%)”.
